# P27 Protects Neurons from Ischemic Damage by Suppressing Oxidative Stress and Increasing Autophagy in the Hippocampus

**DOI:** 10.3390/ijms21249496

**Published:** 2020-12-14

**Authors:** Woosuk Kim, Hyun Jung Kwon, Hyo Young Jung, Kyu Ri Hahn, Yeo Sung Yoon, In Koo Hwang, Soo Young Choi, Dae Won Kim

**Affiliations:** 1 Department of Biomedical Sciences, and Research Institute for Bioscience and Biotechnology, Hallym University, Chuncheon 24252, Korea; tank3430@hallym.ac.kr; 2 Department of Anatomy and Cell Biology, College of Veterinary Medicine, and Research Institute for Veterinary Science, Seoul National University, Seoul 08826, Korea; hyoyoung@snu.ac.kr (H.Y.J.); hkinging@snu.ac.kr (K.R.H.); ysyoon@snu.ac.kr (Y.S.Y.); vetmed2@snu.ac.kr (I.K.H.); 3 Department of Biochemistry and Molecular Biology, Research Institute of Oral Sciences, College of Dentistry, Gangneung-Wonju National University, Gangneung 25457, Korea; donuts25@gwnu.ac.kr

**Keywords:** p27^Kip1^, oxidative stress, ischemia, hippocampus, autophagosome, gerbil

## Abstract

p27^Kip1^ (p27), a well-known cell regulator, is involved in the regulation of cell death and survival. In the present study, we observed the effects of p27 against oxidative stress induced by H_2_O_2_ in HT22 cells and transient ischemia in gerbils. Tat (*trans*-acting activator of transcription) peptide and p27 fusion proteins were prepared to facilitate delivery into cells and across the blood-brain barrier. The tat-p27 fusion protein, rather than its control protein Control-p27, was delivered intracellularly in a concentration and incubation time-dependent manner and showed its activity in HT22 cells. The localization of the delivered Tat-p27 protein was also confirmted in the HT22 cells and hippocampus in gerbils. In addition, the optimal concentration (5 μM) of Tat-p27 was determined to protect neurons from cell death induced by 1 mM H_2_O_2_. Treatment with 5 μM Tat-p27 significantly ameliorated H_2_O_2_-induced DNA fragmentation and the formation of reactive oxygen species (ROS) in HT22 cells. Tat-p27 significantly mitigated the increase in locomotor activity a day after ischemia and neuronal damage in the hippocampal CA1 region. It also reduced the ischemia-induced membrane phospholipids and ROS formation. In addition, Tat-p27 significantly increased microtubule-associated protein 1A/1B light chain 3A/3B expression and ameliorated the H_2_O_2_ or ischemia-induced increases of p62 and decreases of beclin-1 in the HT22 cells and hippocampus. These results suggest that Tat-p27 protects neurons from oxidative or ischemic damage by reducing ROS-induced damage and by facilitating the formation of autophagosomes in hippocampal cells.

## 1. Introduction

Transient forebrain ischemia is one of the most life-threatening neurological diseases, and it causes a decline in quality of life in survivors [1,2]. Ischemia is induced by the interruption of arterial supply to the brain, and reperfusion causes extensive formation of reactive oxygen species (ROS) and intracellular calcium influx in brain tissues [3,4]. Several cell death mechanisms induced by transient forebrain ischemia have been proposed, including excitotoxicity, oxidative stress, and inflammatory responses [5,6]. Finally, affected neurons undergo cell death, such as apoptosis, necroptosis, and autophagy, to maintain homeostasis by reducing the propagation of neuronal death [7].

Cell cycle machinery is believed to play an important role in neuronal death after ischemia. Among cell cycle regulators, p27^Kip1^ (p27) is recognized as a tumor suppressor that negatively regulates cell proliferation and differentiation [8]. However, p27 is involved in the regulation of inflammation, aging, cell death, and cell survival [9,10,11,12]. In 1997, Wang et al. demonstrated that the overexpression of p27 causes cell death in all cell types [13]. However, pathological conditions, such as oxygen-glucose deprivation, reduce p27 expression in neocortical neurons before neuronal death [14]. Treatment with p27 siRNA induces neuronal death in cultured cortical neurons [15], and the knockout of p27 causes more pronounced neuronal degeneration in the mouse hippocampal CA3 region [16]. The overexpression of p27 reduces infarct size after myocardial infarction [17,18] and protects neurons from insults induced by trophic factor deprivation [19]. However, another study reported that p27 overexpression decreases recovery in blood flow after hindlimb ischemia [20].

Most studies have evaluated the heart and hindlimb after ischemia to assess the role of p27 in preventing ischemic damage because p27 could not cross the blood-brain barrier [17,18,20,21]. Cell-penetrating peptides are the most effective translocators of large-sized proteins into intracellular organelles and brain structures. Tat (*trans*-acting activator of transcription) was discovered in human immunodeficiency virus-1, and it is widely used to deliver proteins, DNA phages, and liposomes intracellularly [22,23,24]. In addition, Tat-cargo fusion proteins are successfully delivered into the hippocampus [25], and they show neuroprotective effects against ischemic damage [26,27]. Several studies have demonstrated that the Tat-p27 fusion protein is efficiently delivered to cardiomyocytes, and treatment with Tat-p27 protects cardiomyocytes from myocardial infarction [17,18].

In the present study, we synthesized the Tat-p27 fusion protein and investigated its effects and possible mechanisms against oxidative damage in HT22 mouse hippocampal neuronal cell line and ischemic damage in the gerbil hippocampus.

## 2. Results

### 2.1. Intracellular Delivery of Tat-p27 into HT22 Cells

The synthesized Tat-p27 and Control-p27 were purified, and their expressions were evaluated by Coomassie brilliant blue staining and confirmed by western blot analysis to visualize the His-Tag inserted in the vector. One clear Coomassie brilliant blue-stained band was detected in each lane, and clear western bands of Tat-p27 and Control-p27 were found at 29.3 kDa and 27.7 kDa, respectively.

No significant changes in intracellular protein delivery, as confirmed by polyhistidine western blotting, were detected at various times or concentrations during the Control-p27 treatment. However, the incubation of 0.5 to 5.0 μM Tat-p27 in HT22 cells for 60 min showed a significant concentration-dependent increase in polyhistidine levels. In addition, the incubation of 5.0 μM Tat-p27 in HT22 cells resulted in significant time-dependent increments in polyhistidine levels compared with the control or time-matched Control-p27.

The intracellular locations of the delivered Tat-p27 and Control-p27 proteins were confirmed by immunocytochemical staining. In the 5.0 μM Control-p27-treated HT22 cells, no polyhistidine-positive structures were found, whereas in the 5.0 μM Tat-p27-treated cells, polyhistidine-positive structures were mainly detected in the cytoplasm, but not in the nucleus.

**Figure 1:**
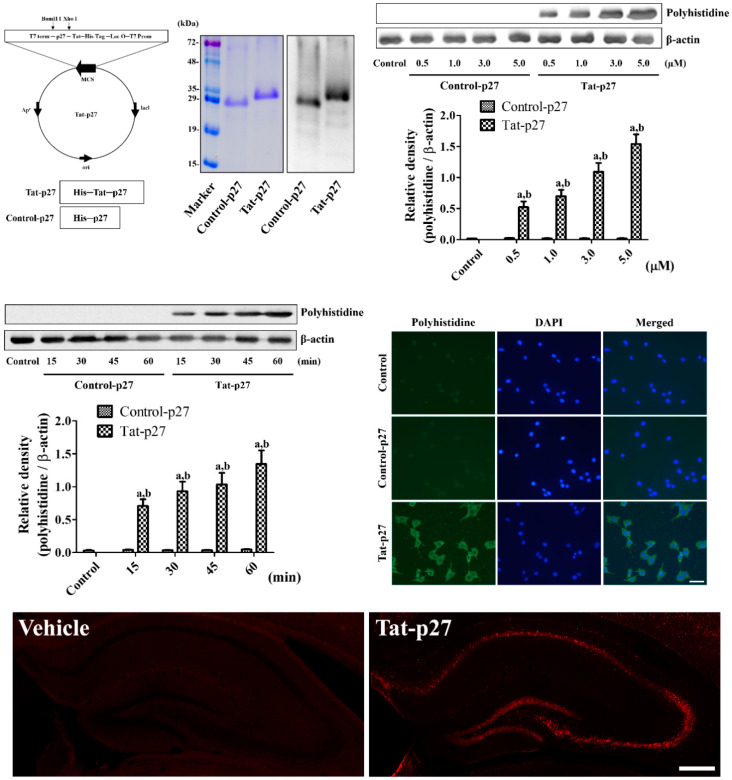
Construction and delivery of Tat-p27 and Control-p27 into HT22 cells. Vectors of Tat-p27 and Control-p27 were constructed and their expressions were confirmed by Coomassie brilliant blue staining and western blot analysis for polyhistidine. Intracellular delivery of Tat-p27 and Control-p27 was confirmed by western blot for polyhistidine using various concentrations (0.5–5.0 μM) and incubation durations (15–60 min) in HT22 cells. Western blot assays were performed in at least triplicate and bar graph represents the mean ± standard deviation. Density of polyhistidine bands was analyzed using two-way analysis of variance (ANOVA) followed by a Bonferroni’s post hoc test (^a^ *p* < 0.05, significantly different from the control group; ^b^ *p* < 0.05, significantly different from the concentration- or time-matched Control-p27 group). Localizations of intracellular-delivered Tat-p27 and Control-p27 were detected by polyhistidine immunocytochemistry in HT22 cells. Scale bar = 20 μm. Visualization of Tat-p27 and Control-p27 delivery into the hippocampus is observed 8 h after protein treatment by polyhistidine immunofluorescent staining. Scale bar = 400 μm.

Delivery of Tat-p27 and Control-p27 proteins were visualized by immunohistochemical staining for polyhistidine 8 h after protein treatment in normal gerbils. In the 3 mg/kg Control-p27-treated gerbils, polyhistidine immunoreactivity was not detected in the hippocampus, while in the 3 mg/kg Tat-p27-treated group, polyhistidine immunoreactivity was abundantly observed in the hippocampus, including the CA1 region (Figure 1).

### 2.2. Effect of Tat-p27 on Oxidatively Stressed HT22 Cells

Concentration-dependent changes of p27 activity was measured after exposure to 1 mM H_2_O_2_ for 1 h using p27^Kip1^ enzyme-linked immunosorbent assay (ELISA) kit. In the H_2_O_2_ alone group, p27 activity was dramatically decreased to 55.6% of that of the control group. Various concentration (0.5 to 5.0 μM) of Control-27 showed similar p27 activity (53.6–55.7% of control) compared to that in the H_2_O_2_ alone group. However, treatment with Tat-p27 increased p27 activity in a concentration-dependent manner and treatment with 5.0 μM Tat-p27 was 91.7% of that of the control group.

Oxidative stress was induced by exposure to 1 mM H_2_O_2_ for 5 h to determine the optimal concentration of Tat-p27 for protecting the neurons from oxidative damage by the water-soluble tetrazolium salt-1 (WST-1) assay. In the H_2_O_2_ alone group, cell viability significantly decreased to 60.5% of that of the control group, and treatment with Control-p27 did not show any significant increase in cell viability after exposure to H_2_O_2_ compared to H_2_O_2_ alone group. Tat-p27 incubation resulted in a concentration-dependent increase in cell viability after exposure to H_2_O_2_. Incubation with 5.0 μM Tat-p27 significantly increased cell viability to 89.3% of that of the control group.

To elucidate the effect of Tat-p27 on DNA fragmentation, the cells were incubated with 5.0 μM Tat-p27 for 1 h and followed by 1 mM H_2_O_2_ for 3 h. In the control group, few terminal deoxynucleotidyl transferase dUTP nick end labeling (TUNEL)-positive cells were found among the HT22 cells. In the H_2_O_2_ alone group, numerous TUNEL-positive cells were detected, and TUNEL fluorescence intensity significantly increased to 497.7% of that of the control group. Treatment with 5.0 μM Control-p27 showed a similar TUNEL fluorescence intensity after exposure to H_2_O_2_ compared to that in the H_2_O_2_ alone group. Incubation with 5.0 μM Tat-p27 decreased the number of TUNEL-positive cells, and the fluorescence intensity was significantly lower (186.5% of that of the control group) than those of the H_2_O_2_ alone and Control-p27-treated groups.

**Figure 2:**
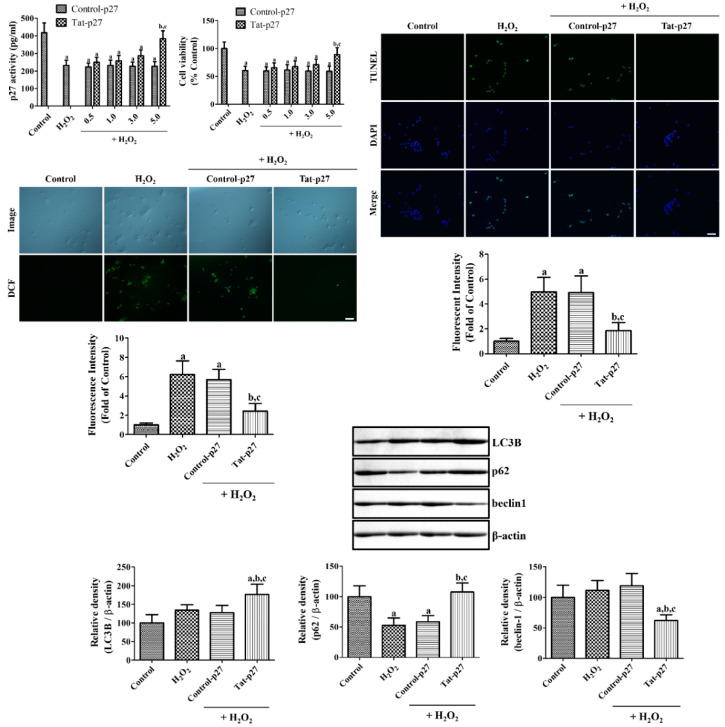
Ameliorative effects of Tat-p27 and Control-p27 on H_2_O_2_-induced oxidative stress in HT22 cells. Cell damage induced by 1 mM H_2_O_2_ and p27 activity of Tat-p27 and Control-p27 was assessed using p27^Kip1^ enzyme-linked immunosorbent assay (ELISA) kit. Cell damage induced by 1 mM H_2_O_2_ and effects of Tat-p27 and Control-p27 were determined using water-soluble tetrazolium salt-1 (WST-1) assay to determine the optimal concentration of Tat-p27. DNA fragmentation induced by 1 mM H_2_O_2_ and effects of Tat-p27 and Control-p27 were visualized with terminal deoxynucleotidyl transferase dUTP nick end labeling (TUNEL) staining following subsequent incubation with 5.0 μM Tat-p27 or Control-p27 for 1 h and 1 mM H_2_O_2_ for 3 h. ROS formation induced by 1 mM H_2_O_2_ and effects of Tat-p27 and Control-p27 were visualized with 2,7-dichlorofluorescein (DCF) fluorescence following subsequent incubation with 5.0 μM Tat-p27 or Control-p27 for 1 h, 1 mM H_2_O_2_ for 10 min, and 20 μM DCF-diacetate (DCF-DA) for 30 min. The intensities of TUNEL-positive cells and DCF fluorescence were also measured. Scale bar = 50 μm. Autophagy was evaluated using western blot for microtubule-associated 1A/1B light chain 3B (LC3B), beclin-1, and p62 1 h after exposure to 1 mM H_2_O_2_ with or without preincubation with Tat-p27 and Control-p27 for 1 h. Western blot assays were performed in at least triplicate and the bar graph represents the mean ± standard deviation. Cell viability, the intensity of fluorescence, and optical density of western bands were analyzed using one-way ANOVA followed by a Bonferroni’s post hoc test (^a^ *p* < 0.05, significantly different from the control group; ^b^ *p* < 0.05, significantly different from the H_2_O_2_ alone group; ^c^ *p* < 0.05, significantly different from the Control-p27-treated group with H_2_O_2_). Bar graph represents the mean ± standard deviation.

ROS formation was assessed by measuring the fluorescence intensity of 2,7-dichlorofluorescein (DCF) converted from DCF diacetate (DCF-DA). In the control group, the DCF fluorescence detected in the HT22 cells was weak. In the H_2_O_2_ alone group, a strong DCF fluorescence was observed in the cytoplasm of the HT22 cells, and the DCF fluorescence intensity significantly increased to 622.7% of that of the control group. The treatment with Control-p27 slightly decreased the DCF fluorescence intensity after exposure to H_2_O_2_, but the difference was not statistically significant. Incubation with Tat-p27 significantly decreased the DCF fluorescence intensity to 242.8% of that of the control group (Figure 2).

H_2_O_2_-induced autophagy was assessed by western blotting for microtubule-associated 1A/1B light chain 3B (LC3B), beclin-1, and p62 in the HT22 lysates 1 h after Tat-p27 and Control-p27 treatment. Treatment with H_2_O_2_ showed a slight increase in LC3B protein levels to 134.3% of the control group, but statistical significance was not detected between control and the H_2_O_2_ alone group. The treatment with Control-p27 showed similar LC3B protein levels compared to the H_2_O_2_ alone group. However, incubation with Tat-p27 showed significant increases in LC3B expression levels to 176.8% of control compared to control, H_2_O_2_ alone, or Control-p27 groups.

p62 protein levels were dramatically decreased in the H_2_O_2_ alone group by 53.1% of control group. The treatment with Control-p27 showed similar levels of p62 compared to that in the H_2_O_2_ alone group. In contrast, incubation of Tat-p27 recovered the reduction of p62 protein levels induced by H_2_O_2_ alone group and showed similar levels of beclin-1 compared to that in the control group. 

There were no significant changes in beclin-1 protein levels between control and H_2_O_2_ alone group. In addition, the treatment with Control-p27 showed similar beclin-1 protein levels compared to that in the control or H_2_O_2_ alone group. However, Tat-p27 treatment showed a significant reduction of beclin-1 protein levels in HT22 cells. 

### 2.3. Effect of Tat-p27 on Cell Death in the Gerbil Hippocampus

Neuronal damage induced by ischemia was assessed using locomotor activity and neuronal nuclei (NeuN) immunohistochemistry 1 day and 4 days after ischemia, respectively. In the Tat peptide- and 3 mg/kg Control-p27-treated groups, the distance traveled significantly increased to 260.1% and 285.7% of that of the control group, respectively. The Tat-p27-treated group showed a dose-dependent decrease in the distance traveled, which was shorter than those of the Tat peptide- and Control-p27-treated groups. The changes in the 1 and 3 mg/kg Tat-p27-treated groups were statistically significant.

**Figure 3:**
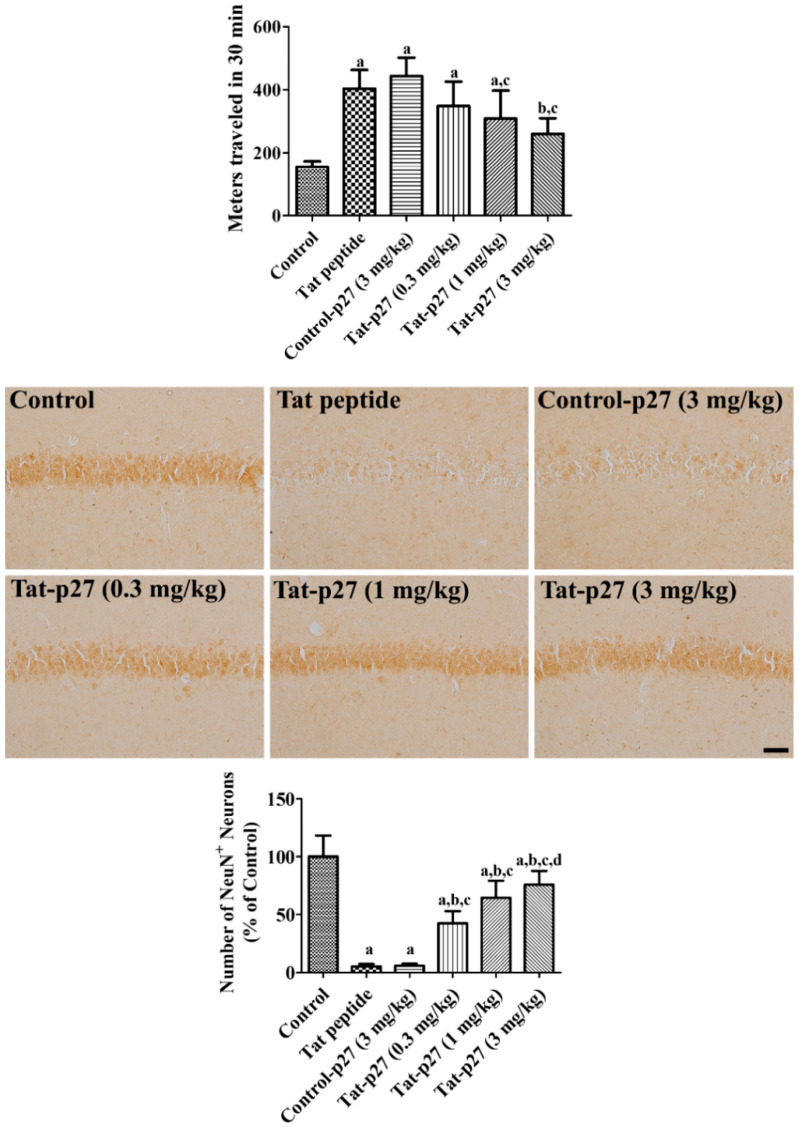
Ameliorative effects of Tat-p27 and Control-p27 on ischemia-induced hyperactivity and cell death in the gerbils. Locomotor activity was recorded for 30 min 1 day after ischemia and the traveled distance was calculated in the sham-operated (control), Tat peptide (vehicle), Control-p27 (3 mg/kg), and Tat-p27 (0.3, 1, and 3 mg/kg) groups. Survived mature neurons were visualized with immunohistochemical staining for NeuN in the hippocampal CA1 region in the control, Tat peptide, Control-p27, and Tat-p27 groups 4 days after ischemia. Scale bar = 50 μm. The traveled distance and the number of NeuN-immunoreactive neurons were analyzed using one-way ANOVA followed by a Bonferroni’s post hoc test (*n* = 5 per group; ^a^ *p* < 0.05, significantly different from the control group; ^b^ *p* < 0.05, significantly different from the Tat peptide group; ^c^ *p* < 0.05, significantly different from the Control-p27 group; ^d^ *p* < 0.05, significantly different from the 0.3 mg/kg Tat-p27 group). Bar graph represents the mean ± standard deviation.

In the control group, several NeuN-immunoreactive neurons were found in the stratum pyramidale of the hippocampal CA1 region. In the Tat peptide- and 3 mg/kg Control-p27-treated groups, only a few NeuN-immunoreactive neurons were detected in the stratum pyramidale of the hippocampal CA1 region, whereas they were abundant in the other regions; the number of NeuN-immunoreactive neurons was 5.2% and 5.8% of that of the control group, respectively. In the Tat-p27-treated groups, several NeuN-immunoreactive neurons were found in the stratum pyramidale of the CA1 region. This group showed a dose-dependent increase in the NeuN-immunoreactive neurons, which was significantly higher in the 0.3 (42.5% of control), 1 (64.5% of control), and 3 mg/kg (75.8% of control) Tat-p27-treated group than in the Tat peptide-treated group (Figure 3).

### 2.4. Mechanisms of Tat-p27 against Ischemic Damage in Gerbils

To elucidate the possible neuroprotective mechanisms of Tat-p27 against ischemia, the levels of oxidative products such as malondialdehyde (MDA) and 8-iso-prostaglandin F2α (8-iso-PGF2α) were measured 3 and 12 h after ischemia in the hippocampus. The MDA levels were significantly higher in the Tat peptide- and Control-p27-treated groups (273.4% and 198.9%, respectively) than in the control group 3 h and 12 h after ischemia. The MDA levels were significantly lower in the Tat-p27-treated group than in the Tat peptide- and Control-p27-treated groups (149.1% and 120.4% of that of the control group, respectively). The levels of 8-iso-PGF2α in the Tat peptide-treated group significantly increased to 210.0% and 548.9% of that of the control group 3 h and 12 h after ischemia, respectively. The levels of 8-iso-PGF2α were lower in the Control-p27-treated group than in the Tat peptide-treated group, and statistical significance was observed at 12 h, not 3 h, after ischemia. The 8-iso-PGF2α levels of the Tat-p27-treated group decreased to 127.7% and 173.5% of that of the control group 3 and 12 h after ischemia, respectively.

ROS formation in the hippocampus was visualized by dihydroethidium (DHE) staining 12 h after ischemia when the oxidative adducts increased in the hippocampus. In the control group, DHE fluorescence was not detectable in the hippocampal CA1 region, whereas in the Tat peptide- and Control-p27-treated groups, DHE fluorescence was observed in the pyramidal cells of the CA1 region. In these groups, DHE fluorescence intensity significantly increased to 181.9% and 169.9% of that of the control group. In the Tat-p27-treated group, DHE fluorescence intensity significantly decreased to 123.5% of that of the control group.

Autophagy was validated by western blotting for LC3A/B, beclin-1, and p62 in the hippocampus 12 h after ischemia. LC3A/B protein levels in the Tat peptide- and Control-p27-treated groups significantly increased to 141.9% and 140.0% of that of the control group, respectively. In the Tat-p27-treated group, the LC3A/B protein levels significantly increased to 185.0% of that of the control group. 

p62 protein levels were significantly decreased in the Tat peptide- and Control-p27-treated groups to 57.1% and 58.5% of that of the control group, respectively. In the Tat-p27-treated group, p62 protein levels were significantly increased compared to that in the Tat peptide- and Control-p27-treated groups and showed similar levels to 87.7% of that of the control group.

**Figure 4:**
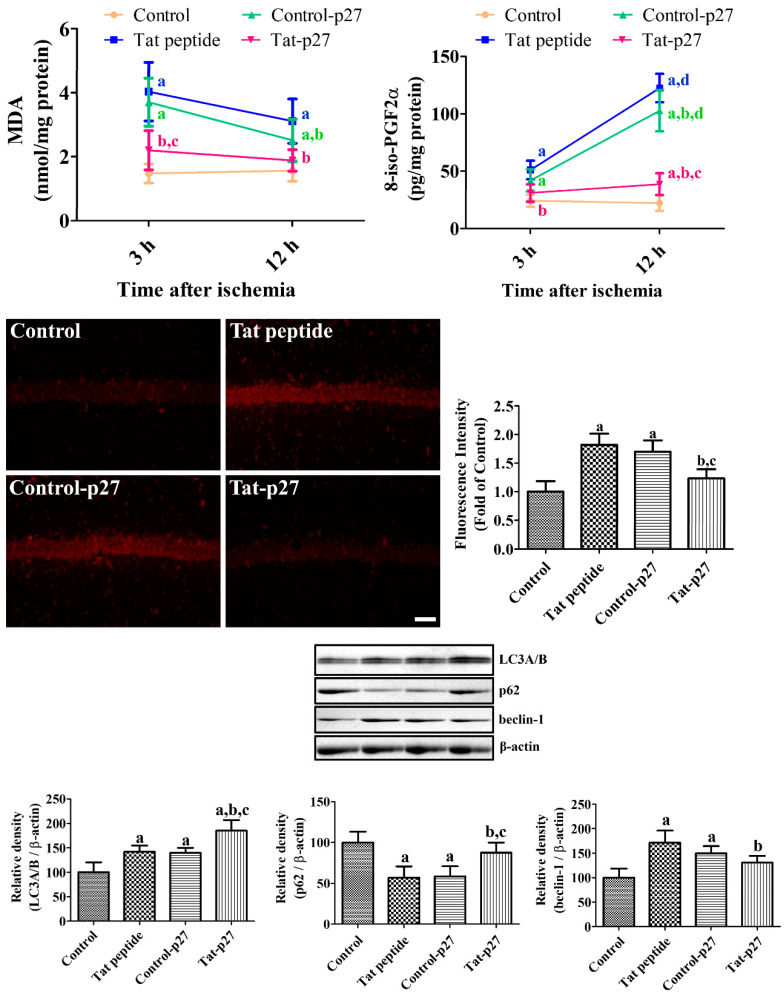
Ameliorative effects of Tat-p27 and Control-p27 on ischemia-induced oxidative stress and autophagy. Reactive oxygen species (ROS)-induced malondialdehyde (MDA) and 8-iso-prostaglandin F2α (8-iso-PGF2α) levels were measured using enzyme-linked immunosorbent assay (ELISA) assay kits 3 and 12 h after ischemia in the Tat peptide, Control-p27, and Tat-p27 groups. ROS were visualized with dihydroethidium (DHE) fluorescence in the hippocampal CA1 region 3 and 12 h after ischemia in these groups. Scale bar = 50 μm. Autophagy was evaluated using western blot for microtubule-associated 1A/1B light chain 3A/B (LC3A/B), beclin-1, and p62 12 h after ischemia. Data were analyzed using one-way or two-way ANOVA followed by a Bonferroni’s post hoc test (*n* = 5 per group; ^a^ *p* < 0.05, significantly different from the control group; ^b^ *p* < 0.05, significantly different from the Tat peptide group; ^c^ *p* < 0.05, significantly different from the Control-p27 group; ^d^ *p* < 0.05, significantly different from 3 h post-ischemic group). Bar graph represents the mean ± standard deviation.

Beclin-1 protein levels were significantly increased in the Tat peptide- and Control-p27-treated groups to 171.3% and 149.8% of that of the control group, respectively. In the Tat-p27-treated group, beclin-1 protein levels were significantly decreased compared to Tat peptide-treated group and were 130.6% of the control group (Figure 4). 

## 3. Discussion

Cell cycle machinery is considered to be the most important mechanism underlying neuronal death after brain damage [28]. p27^Kip1^, a cell cycle modulator, is considered as a key regulator of cell entry, and it plays a pivotal role in autophagy [29,30,31]. Autophagy is believed to be important for cell death, and it is induced under normal and pathological conditions [32,33]. In addition, ROS are closely related to the regulation of autophagy [34]. In the present study, we facilitated the easy crossing of the cell membrane and blood-brain barrier by the Tat-p27 fusion protein to understand the role of p27 in oxidative or ischemic damage. The purified Tat-p27 and Control-p27 were subjected to Coomassie staining and western blot for polyhistidine at approximately 29.3 kDa and 27.7 kDa because of the molecular weights of His-tag (0.7 kDa) and the Tat peptide (1.6 kDa). Tat-p27 was efficiently delivered into HT22 cells in a concentration- and time-dependent manner. The delivered Tat-p27 protein was visualized by immunostaining for polyhistidine in HT22 cells and gerbil hippocampus because the vector consisted of p27 protein, Tat peptide, and His-tag. This result suggests that the Tat-p27 fusion protein was successfully constructed and the p27 protein was efficiently introduced intracellularly into the HT22 cells with a concentration- and time-dependent manner, as well as into the hippocampus 8 h after treatment. This is consistent with reports of previous studies that Tat fusion proteins can be delivered to targeted cells, including hippocampal cells [26,27], glial cells [35], and cardiomyocytes in a concentration-dependent manner [36].

Several studies have demonstrated that oxidative stress induced by H_2_O_2_ reduces p27 levels in various cell lines [37,38]. In addition, glutamate decreases p27 expression in primary neuronal cultures [15]. In the present study, we treated HT22 cells exposed to 1 mM H_2_O_2_ with Tat-p27 and its control (Control-p27) to induce oxidative damage, elucidate the effects of Tat-p27, and determine the optimal concentration of Tat-p27 for preventing cellular damage induced by H_2_O_2_ in HT22 cells. First of all, we observed the decreases of p27 activity after exposure to H_2_O_2_ and the treatment with Tat-p27, not Control-p27, ameliorated the reduction of p27 activity with concentration-dependent manner. This result suggests that transduced Tat-p27 shows its functions in the HT22 cells. Cell viability was assessed using the WST-1 assay, which is reliable for monitoring cell viability [39]. Cell viability significantly decreased after H_2_O_2_ treatment and increased after treatment with 5.0 μM Tat-p27, but not Control-p27 treatment. In gerbils, increased motor activity was observed after transient forebrain ischemia because of neuronal damage in the hippocampus [40]. Neuronal death was also observed in the hippocampal CA1 region 4 days after ischemia. In the present study, treatment with Tat-p27 significantly ameliorated hyperactivity and neuronal death in the hippocampal CA1 region. This suggests that Tat-p27 protects neurons from ischemic damage in the hippocampal CA1 region. This result is consistent with a previous study that reported that Tat-p27 ameliorates apoptotic neuronal death via glucose deprivation in cardiomyocytes [18] and protects cells from myocardial infarction [17]. The overexpression of p27 protects neurons from trophic factor deprivation [19] and DNA damage [41], whereas the suppression of p27 using siRNA causes cell death in cultured cortical neurons [42]. In p27 knockout mice, neuronal damage is more pronounced in the hippocampal CA3 region than in the wild-type mice [16]. However, the overexpression of p27 decreases the circulation of blood flow in the hindlimb after ischemia [20], whereas the knockout of p27 facilitates recovery by the formation of collateral circulation in the hindlimb [21]. Treatment with p27 siRNA also mitigated the apoptosis of cortical neurons induced by Aβ_42_ [43]. The discrepancies in the roles of p27 may be associated with the cellular and animal models used in the study.

In this study, we also observed the effects of 5.0 μM Tat-p27 and Control-p27 on DNA fragmentation and ROS formation using TUNEL and DCF fluorescence. Pre-incubation with Tat-p27 significantly reduced DNA fragmentation and ROS formation induced by H_2_O_2_. This result is consistent with the report of a previous study that glucose deprivation induces cell death in rat neonatal cardiomyocytes and treatment with Tat-p27 protects cardiomyocytes from cell damage induced by glucose deprivation [18]. In addition, the overexpression of p27 induced by adenoviral vector 9 reduced TUNEL-positive cells in the cardiomyocytes of lipopolysaccharide-treated rats [44]. We also confirmed the antioxidative potential of ethidium and the fluorescence of 2-hydroxyethidium, which was generated from DHE by oxidation [45] in Mongolian gerbils 12 h after ischemia. Treatment with Tat-p27 significantly reduced the ischemia-induced formation of E^+^ and the fluorescence of 2-OH-E^+^ as well as peroxidation products such as MDA and 8-iso-PGF2α, indicating that Tat-p27 reduces ROS formation induced by ischemia/reperfusion in the hippocampal CA1 region. However, the hearts of aged p27 knockout mice showed lower DNA adducts after ovariectomy than those of wild-type mice [46]. 

To elucidate the possible mechanisms of Tat-p27 against oxidative stress and ischemic damage, western blotting for LC3A/B (or LC3B), p62, and beclin-1 was conducted because ROS was closely related to the regulation of autophagy [34]. In the present study, we observed oxidative stress induced by H_2_O_2_ moderately increases LC3A/B levels and transient forebrain ischemia increases LC3 expression, indicating the promotion of autophagy by oxidative stress and ischemia. This result is consistent with previous reports showing that LC3B expression is increased after ischemia [47,48] and hypoxic-ischemic injury [49,50,51]. In the present study, we observed that treatment with Tat-p27 significantly increased LC3A/B levels in the hippocampus, indicating that Tat-p27 increases autophagy to facilitate the modulation of cellular conditions because an imbalance in autophagic flux can lead to neuronal damage [52]. Beclin-1 is involved in the initiation of autophagosome formation and its cleavage by several caspases inactivates the autophagy, and promotes apoptosis [53]. Knockdown of beclin-1 prevented neuronal damage and neurological deficits in rats from focal ischemia [54]. In contrast, p62 is one of adaptor proteins in autophagy and several studies demonstrated the negative correlation between p62 and autophagy level [55,56]. In the present study, we observed significant decreases of p62 levels in HT22 cells after exposure to H_2_O_2_ and in the gerbil hippocampus after ischemia. In contrast, beclin-1 levels were significantly increased in the gerbil hippocampus, not in the HT22 cells exposed to H_2_O_2_. This discrepancy may be associated with the potency of stress induced by deprivation of glucose and oxygen (ischemia) and oxidative stress. Several studies demonstrated the cerebral ischemia induced the excessive autophagy and it caused neuronal death [57,58]. However, in the present study, we sacrificed the animals 24 h after ischemia, but the neuronal death is not occurred in this time. The increases of autophagy may be compensatory mechanisms to scavenge the damaged cells in early time period of ischemia and it could prevent neuronal death in the hippocampal CA1 region. This result is consistent with a previous study that Tat-p27 prevented apoptotic cell death by promoting autophagy in metabolically stressed cardiomyocytes and in myocardial infarction [18].

In conclusion, Tat-p27 protects neurons from oxidative and ischemic damage in the gerbil hippocampus, possibly reducing oxidative damage and facilitating autophagy. These results suggest that Tat-p27 can be used to reduce neuronal damage induced by oxidative and ischemic stress.

## 4. Materials and Methods

### 4.1. Synthesis of Tat-p27 and Validation of Intracellular Delivery of p27 into HT22 Cells and Gerbil Brain

Tat-p27 and Control-p27 were synthesized by cloning human p27 cDNA in a TA vector with or without the Tat-1 expression vector, respectively. In addition, a polyhistidine-tag was inserted into the vectors to visualize the intracellular delivery of p27. Xho I has a CTC GAG sequence (leucine glutamic acid), which connects the Tat peptide and p27 protein. Tat-p27 and Control-p27 plasmids were amplified, as described in a previous study [59], and the purified proteins were obtained and validated by Coomassie brilliant blue staining and western blotting using polyhistidine antibody (1:3000, Sigma, St. Louis, MO, USA). Western bands were visualized with chemiluminescent reagents according to the manufacturer’s protocol (Amersham, Franklin Lakes, NJ, USA). Western blot assays were performed in at least triplicate in each experiment.

To assess the concentration- and time-dependent delivery of Tat-p27 and Control-p27 in HT22 cells, various concentrations (0.5 to 5 μM) of proteins were incubated for 60 min or 5 μM proteins were added to HT22 cells for various durations (15 to 60 min). Thereafter, the cells were harvested and intracellular delivery was observed by western blot for polyhistidine, as described in a previous study [26,27]. In addition, intracellular delivery of proteins was visualized by immunocytochemical staining for polyhistidine. Briefly, 5 μM of proteins were incubated in HT22 cells for 60 min, and the cells were fixed with 4% paraformaldehyde for 10 min at 25 °C. Thereafter, the cells were incubated with mouse anti-polyhistidine antibody (1:2000, Sigma, St. Louis, MO, USA) for 2 h at 25 °C and Alexa Fluor^®^ 488-conjugated anti-mouse IgG (1:1000; Jackson ImmunoResearch, West Grove, PA, USA) for 1 h at 25 °C with 1 μg/mL 4,6-diamidino-2-phenylindole (DAPI, Thermo Fisher Scientific, Waltham, MA, USA). Immunoreactive structures were taken with a confocal fluorescence microscope (LSM 510 META NLO; Zeiss GmbH, Jena, Germany).

To measure p27 activity in the HT22 cells exposed to H_2_O_2_ with Tat-p27 and Control-p27, various concentrations (0.5 to 5 μM) of proteins were incubated for 60 min. Thereafter, the cells were exposed to 1 mM H_2_O_2_ for 1 h to induce cellular damage in HT22 cells, and p27 activity was assessed in the cell lysates using mouse p27^Kip1^ SimpleStep^®^ ELISA kit (Abcam, Cambridge, UK) according to instructor’s protocol.

The animals used (Mongolian gerbils, male, 3-month old) were obtained from Japan SLC Inc. (Shizuoka, Japan). Animal use and experimental protocols were approved by the Institutional Animal Care and Use Committee (IACUC) of Seoul National University (SNU-190516-7). To validate the intracellular delivery of Tat-p27 and Control-p27 in HT22 cells, animals were sacrificed with 5% isoflurane (Hana Pharm Co., Ltd., Hwaseong, South Korea) anesthesia 8 h after intraperitoneal treatment of 3 mg/kg Tat-p27 and Control-p27 protein. Thereafter, transcardiac perfusion was performed with physiological saline and 4% paraformaldehyde. The brain was quickly removed from the skull and trimmed between 2.0 and 2.7 mm caudal to the bregma based on a gerbil brain atlas [60]. Trimmed brain slices were sectioned coronally, and serial 30-μm sections were collected in 6-well plates. Seven sections per animals separated from each other by 90-μm were selected and incubated with mouse anti-polyhistidine antibody (1:500; Sigma, St. Louis, MO, USA). The sections were visualized by reacting with Cy3-conjugated anti-mouse IgG (1:600; Jackson ImmunoResearch, West Grove, PA, USA).

### 4.2. Effect and Mechanism of Tat-p27 and Control-p27 against Oxidative Damage Induced by H_2_O_2_ in HT22 Cells

To assess the concentration-dependent effect of Tat-p27 and Control-p27 against oxidative damage and determine the optimal concentration for neuroprotection, HT22 cells were incubated with various concentrations (0.5 to 5.0. µM) of the proteins for 1 h. Thereafter, the cells were exposed to 1 mM H_2_O_2_ for 5 h to induce cellular damage in HT22 cells, and the WST-1 test was performed to detect cell damage using the WST-1 assay kit (Abcam, Cambridge, UK) following the manufacturer’s protocol.

To elucidate the role of Tat-p27 and Control-p27 on DNA fragmentation in HT22 cells after oxidative stress, HT22 cells were sequentially incubated with 5.0 µM proteins for 1 h and 1 mM H_2_O_2_ for 3 h. To evaluate the formation of ROS, the cells were sequentially incubated with 5.0 µM proteins for 1 h, 1 mM H_2_O_2_ for 10 min, and 20 μM DCF-DA for 30 min because ROS formation occurred in the early period after oxidative stress was induced by H_2_O_2_. Thereafter, the cells were fixed with 4% paraformaldehyde for 10 min at 25 °C, and TUNEL staining was performed following the manufacturer’s instructions (Sigma-Aldrich, St. Louis, MO, USA). ROS formation was detected by observing the fluorescence of DCF, which was converted from DCF-DA by ROS. The fluorescence of TUNEL- and DCF-positive structures was quantified using a Fluoroskan ELISA plate reader (Labsystems Oy, Helsinki, Finland).

To elucidate the mechanisms of Tat-p27 and Control-p27 against oxidative damage, HT22 cells were incubated with 5.0 µM of the proteins for 1 h and thereafter, the cells were exposed to 1 mM H_2_O_2_ for 5 h to induce cellular damage in HT22 cells. Western blot analysis was conducted as described in the previous study [59] and western bands were visualized with chemiluminescent reagents according to the manufacturer’s protocol (Amersham, Franklin Lakes, NJ, USA). Primary antibodies were purchased from Sigma for p62, Cell Signaling Technology (Beverly, MA, USA) for beclin-1, and Abcam for the LC3A/B antibody. Western blot assays were performed in at least triplicate in each experiment.

### 4.3. Effect of Tat-p27 and Control-p27 on Brain Ischemia in Gerbils

To induce transient forebrain ischemia, the animals were anesthetized with 3% isoflurane (Hana Pharm Co., Ltd., Hwaseong, South Korea) and common carotid arteries were isolated through a ventral midline incision. Both common carotid arteries were occluded with non-traumatic clips for 5 min, and the lack of blood flow was confirmed by observing the central artery of the retina using an ophthalmoscope (HEINE K180^®^, Heine Optotechnik, Herrsching, Germany). Thereafter, the clips were removed, and Tat-p27 (0.3, 1, and 3 mg/kg), and Control-p27 (3 mg/kg) were administered to the gerbils 30 min after reperfusion.

To assess the ischemia-induced increase in locomotor activity the day after ischemia, traveling activity was recorded with a digital camera system (Basler106200, Ahrensburg, Germany) for 30 min. The locomotor activity was analyzed using Ethovision XT14 (Noldus, Wageningen, The Netherlands), as described in previous studies [26,27].

Neuronal damage by ischemia was confirmed, and the optimal concentration of Tat-p27 was determined by immunohistochemical analysis of NeuN 4 days after ischemia, as described in previous studies [26,27]. Briefly, animals were re-anesthetized with 5% isoflurane (Hana Pharm Co., Ltd., Hwaseong, South Korea), and transcardiac perfusion was performed with physiological saline and 4% paraformaldehyde. The brain was quickly removed from the skull and trimmed between 2.0 and 2.7 mm caudal to the bregma based on a gerbil brain atlas [60]. Trimmed brain slices were sectioned coronally, and serial 30-μm sections were collected in 6-well plates. Seven sections per animals separated from each other by 90-μm were selected and incubated with mouse anti-NeuN antibody (1:1000; EMD Millipore, Temecula, CA, USA), biotinylated goat anti-mouse IgG (1:200; Vector, Burlingame, CA, USA), and Vectastain^®^ ABC kits (Vector, Burlingame, CA, USA). The sections were visualized by reacting with 3,3′-diaminobenzidine tetrachloride (Sigma, St. Louis, MO, USA).

### 4.4. Mechanism of Tat-p27 and Control-p27 on Brain Ischemia in Gerbils

To elucidate the possible mechanisms of Tat-p27 against ischemic damage, transient forebrain ischemia was induced as described above, and Tat peptide, Control-p27 (3 mg/kg), and Tat-p27 (3 mg/kg) were administered to the gerbils 30 min after ischemic surgery. The animals were sacrificed 3 h and 12 h after ischemia to measure the oxidative products, such as MDA and 8-iso-PGF2α. In addition, the animals were sacrificed 24 h after ischemia to detect changes in LC3A/B protein, which is a structural protein in the autophagosome membrane in the hippocampus. Briefly, animals were re-anesthetized with 5% isoflurane (Hana Pharm Co., Ltd., Hwaseong, South Korea), and the hippocampi were obtained from 2.0–2.7 mm caudal to the bregma, based on a gerbil brain atlas [60]. Oxidative products were measured using commercially available assay kits such as MDA (Cayman Chemical Company, Ann Arbor, MI, USA) and 8-iso-PGF2α (Cayman Chemical Company). LC3A/B, p62, and beclin-1 protein levels were measured by western blot analysis using rabbit anti-LC3A/B (1:1000; Abcam, Cambridge, UK), rabbit anti-p62 (1:1000; Sigma, St. Louis, MO, USA), and rabbit anti-beclin-1 (1:1000; Cell Signaling, Beverly, MA, USA) antibodies as described above.

In addition, the effects of Tat-p27 on ROS formation in the hippocampal CA1 region were evaluated by histochemical staining for DHE, as described by Wang and Zou [45]. Briefly, animals were re-anesthetized with 5% isoflurane (Hana Pharm Co., Ltd., Hwaseong, South Korea) 12 h after ischemia, and the hippocampi were obtained from 2.0–2.7 mm caudal to the bregma, based on a gerbil brain atlas [53]. Seven sections (30-μm thickness) per animal, separated from each other by 90-μm, were selected and mounted onto gelatin-coated slides. The sections were incubated with 5 μM DHE solution for 20 min at 25 °C and coverslipped with mounting media (Vector, Burlingame, CA, USA).

### 4.5. Data Quantification and Statistical Analysis

The number of NeuN-positive cells was counted in seven sections, which were 90 µm apart, for each animal under ×100 magnification at the mid-point of the hippocampal CA1 region and the entire dentate gyrus, using OPTIMAS 6.5 software (CyberMetrics Corporation, Phoenix, AZ, USA), as described previously [26,27]. DHE fluorescence intensity was quantified by optical density measured using ImageJ software version 1.50 (National Institutes of Health, Bethesda, MD, USA), as described previously [27]. DHE reactivity in the dentate gyrus was measured, and the unlabeled structures were subtracted. Grayscale (0 to 255) and pixel numbers in the DHE reactive structures were multiplied and total optical densities were obtained. The data are presented as percent averages with standard deviation (which was set as 100%), and the differences in averages were statistically analyzed by a one-way or two-way analysis of variance (ANOVA) followed by Bonferroni’s post hoc test using GraphPad Prism 5.01 software (GraphPad Software, Inc., La Jolla, CA, USA).
